# VEGF-A/VEGFR-2 and FGF-2/FGFR-1 but not PDGF-BB/PDGFR-β play important roles in promoting immature and inflammatory intraplaque angiogenesis

**DOI:** 10.1371/journal.pone.0201395

**Published:** 2018-08-20

**Authors:** Yang Mao, Xiaoqiong Liu, Yu Song, Chungang Zhai, Lei Zhang

**Affiliations:** 1 Key Laboratory of Cardiovascular Remodeling and Function Research, Chinese Ministry of Education and Chinese Ministry of Health, Qilu Hospital of Shandong University, Jinan, Shandong, China; 2 Department of Traditional Chinese Medicine, Qilu Hospital of Shandong University, Jinan, P.R. China; 3 Department of Cardiology, Shanghai Institute of Cardiovascular Diseases, Zhongshan Hospital, Fudan University, Shanghai, China; University of South Alabama Mitchell Cancer Institute, UNITED STATES

## Abstract

Various angiogenic factors have been shown to play important roles in intraplaque angiogenesis, while little is known about the dynamic expression change and interplay between various angiogenic factors and intraplaque angiogenesis under high cholesterol conditions. New Zealand rabbits underwent balloon injury of the abdominal artery and then were assigned to a control group (n = 15, normal chow) or high cholesterol group (n = 25, 1% high cholesterol diet). At weeks 4, 6, 8, 10, and 12 after acclimation, rabbits (high cholesterol group, n = 5; control group, n = 3) were euthanized. No lesions were observed in the control group. From week 4 to week 12, the expression of vascular endothelial growth factor A (VEGF-A), VEGF receptor 2 (VEGFR-2), fibroblast growth factor 2 (FGF-2), FGF receptor 1 (FGFR-1), platelet-derived growth factor-BB (PDGF-BB), and tumor necrosis factor alpha (TNF-α), the vulnerability index (VI) and the microvessel density (MVD) were significantly elevated in the high cholesterol group; however, PDGF receptor β (PDGFR-β) expression showed little change. Analysis by double-label immunofluorescence (CD31 and Ng2) and FITC-dextran indicated that the neovessels within the plaque were leaky due to a lack of pericytes. As indicated by Pearson’s correlation analysis, there was a highly positive correlation between the VI, MVD, macrophage content, and TNF-α level, and the levels of VEGF-A/VEGFR-2 and FGF-2/FGFR-1. However, no correlations were observed between PDGFR-β levels and the VI or MVD. High expression of VEGF-A/VEGFR-2 and FGF-2/FGFR-1 but not of PDGF-BB/PDGFR-β may contribute to immature and inflammatory intraplaque angiogenesis and plaque instability in a rabbit model of atherosclerosis.

## Introduction

Vulnerable atherosclerotic (AS) plaques are prone to rupture and are a major cause of acute coronary syndrome (ACS). Vulnerable atherosclerotic plaques are characterized by enlarged lipid cores containing an increased number of vasa vasorum and inflammatory cells and have been shown to cause frequent intraplaque hemorrhaging [1].

Under normal conditions, the blood supply and oxygen supply of arterial walls are provided by vasa vasorum-derived microvessels that only penetrate the adventitia. As plaque progression, plaques require extra nutrients in the blood supply, which is predominantly derived from intraplaque neovascularization [2]. Intraplaque angiogenesis refers to the process of new vessel formation within the plaque. Neovessels within the plaque are considered conduits of erythrocytes, lipid, and leukocytes into plaques, which contribute to the deterioration of interendothelial junctions, and thus, intraplaque angiogenesis constitutes a strong indicator of rapid plaque progression and a possible contributing factor to the risk of rupture [3].

Angiogenesis is induced by various growth factors, such as the vascular endothelial growth factor (VEGF) family, fibroblast growth factor (FGF) family, and platelet-derived growth factor (PDGF) family. Among these growth factors, VEGF-A, FGF-2, and PDGF-BB, and their relevant receptors (VEGF receptor 2, VEGFR-2; FGF receptor 1, FGFR-1; and PDGF receptor β, PDGFR-β) play prominent roles in angiogenesis. While various studies including in vitro studies and tumor models have focused on the relationship between these growth factors, no study has directly focused on the role of growth factors in AS plaques. In our previous study, we found that, from week 4 to 12, the expression of VEGF-A and matrix metalloproteinases (MMPs), MMP-1, MMP-2, MMP-3, and MMP-9 increased with AS plaque development and that the expression of MMP-14 substantially decreased [4]. However, the effect of different growth factors on the different stages of atherosclerosis was unclear. The present study was undertaken to investigate possible in vivo associations between the different growth factors and intraplaque angiogenesis under high cholesterol conditions in a rabbit model of atherosclerosis. In this study, we observed a dynamic expression trend of these growth factors and receptors from 4 to 12 weeks after balloon injury under severe hypercholesterolemic conditions.

## Materials and methods

### Animal model

In this study, all experiments complied with the Guide of the Care and Use of Laboratory Animals of the Chinese Ministry of Health (documentation 55, 2001), and the experimental protocol was approved by the Animal Care Committee of Shandong University. Adult male New Zealand white rabbits (n = 40) weighing 1.7–2.3 kg were obtained from Jinan Xilingjiao Culture and Breeding Center (Jinan, Shandong Province, China). Animals were randomly selected and housed at the Animal Care Center of Qilu Hospital of Shandong University.

Following acclimation for 2 weeks, all rabbits (n = 40) underwent balloon-induced endothelial injury of the abdominal aorta as previously described [5, 6] and then were assigned to a control group (n = 15, normal chow) or high cholesterol group (n = 25, 1% high cholesterol diet). The week after the two-week acclimation period was defined as week 0. After balloon injury of the abdominal aorta, every 2 weeks from week 4 to week 12 (weeks 4, 6, 8, 10, and 12), which corresponded to the completion of the animal model, rabbits (high cholesterol group, n = 5; normal chow group, n = 3) were euthanized (phenobarbital 3%) and aortic tissues were harvested.

### Biochemical studies

After the rabbits were fasted overnight, blood samples were collected; then, the rabbits were euthanized. Serum levels of total cholesterol (TC), low-density lipoprotein cholesterol (LDL-C), high-density lipoprotein cholesterol (HDL-C), and triglycerides (TG) were measured by enzymatic assays using an automated biochemical analyzer (Roche Hitachi 917; Block Scientific, NY, USA).

### Histopathological and immunohistochemical (IHC) staining

For histopathological and IHC analyses, abdominal aortic segments from each group were obtained 4, 6, 8, 10, and 12 weeks after the operation. Immunohistochemical staining involved standard techniques as previously described [7]. In brief, endogenous peroxidase activity was inhibited by incubation with 3% H_2_O_2_. Sections were blocked with 5% goat serum in phosphate-buffered saline (PBS) and were incubated overnight at 4°C with primary antibodies against the following; VEGF-A (JH121, Abcam), VEGFR-2 (NB110-60967, Novus), FGF-2 (PAB7972, Abnova), FGFR-1 (ab829, Abcam), PDGF-BB (ab34074, Abcam), PDGFR-β (NB110-60970, Novus), α-actin (ZM0003, ZSGB-BIO), macrophages (RAM-11, M0633, Dako), TNF-α (ab8348, Abcam), and CD31 (ab24590, Abcam). After washing with PBS, the sections were incubated with secondary antibody at 37°C for 30 min. Finally, IHC staining was visualized using a diaminobenzidine kit (Zhongshan Golden Bridge Biotechnology, Beijing, China) according to the manufacturer's instructions. The nucleus was counterstained with hematoxylin (blue). In preliminary experiments, we tested the cross-reactivity between antibodies and rabbit antigens or nonimmune IgG, which was used in negative-control experiments.

### Histological and immunohistochemical analysis

Slides were imaged under a microscope (Olympus BX51; Olympus, Tokyo, Japan), and histopathological parameters were analyzed using a computer-assisted morphometric analysis system (Image-Pro Plus 7.0; Media Cybernetics, Bethesda, MD). Fibrous cap thickness and intima-media thickness (IMT) of aortic plaques were measured at five equidistant points around the cap in each slice; three slices per segment were measured and the values were averaged. Areas with positive staining of VEGF-A, VEGFR-2, FGF-2, FGFR-1, PDGF-BB, PDGFR-β, smooth muscle cells (SMCs), TNF-α, and macrophages (RAM-11) were expressed as an integrated optical density (IOD) of the stained area divided by the plaque area in at least five high-power fields (×200 or ×400). Neovascularization density on digitized images was quantified by counting the total number of CD31-positive microvessels per ×200 microscopic field [8]. The vulnerability index (VI) was calculated as follows: (% macrophage staining + % lipid staining)/(% SMC + % collagen fiber) [9].

### Immunofluorescence staining

To evaluate the distribution of endothelial cells, pericyte cells, PDGFR-β, and macrophages within the AS plaques, slides were blocked in goat serum albumin for 15 minutes. Slides were subsequently incubated with CD31 (ab24590, Abcam), Ng2 (Ab5320, Millipore) PDGFR-β (NB110-60970, Novus) or CD68 (ab201340, Abcam) at 4°C overnight. Sequentially, slides were incubated with secondary antibodies for 1 hour at room temperature. After being washed briefly in PBS, DAPI was applied. Fluorescent images were visualized under a confocal laser-scanning microscope (Leica, Brunswick, Germany).

### Western blot analysis

Proteins were extracted from rabbit aortas. Tissues were lysed in lysis buffer (100 mM Tris-Cl, pH 6.8, 4%(m/v) SDS, 20% (v/v) glycerol, 200 mM β-mercaptoethanol, 1 mM PMSF, and 1 g/ml aprotinin) and proteins were transferred to PVDF membranes (0.45 mm, Millipore), which were then incubated overnight at 4°C with primary antibodies for VEGF-A (1:200, Abcam), VEGFR-2 (1:200, Abcam), FGF-2 (1:500, Novus), FGFR-1 (1:500, Novus), PDGF-BB (1:200, Abcam), PDGFR-β (1:200, Abcam), and β-actin (1:1000, Cell Signaling Technology). Protein bands on the membrane were visualized using chemiluminescence (Millipore) and were quantified by densitometry.

### Permeability of intraplaque neovessels to FITC-dextran

Intraplaque neovessel permeability was evaluated by measuring FITC-dextran accumulation in the plaque. Rabbits were anaesthetized with 3% pentobarbital, and FITC-dextran (4kDa, Sigma) (15 mg/ml in saline) was injected into the ear vein of rabbits using an injector at a dosage of 1 mg/kg [7]. After 10 min, the abdominal plaques were excised, quickly rinsed three times with PBS, and then homogenized in 500 μl of PBS. Next, the medium was centrifuged at 12,000 × g for 10 min at 4 °C. Supernatants were collected to measure FITC fluorescence using a microplate reader (Molecular Devices, Sunnyvale, CA) with an excitation wavelength of 485 nm and an emission wavelength of 538 nm.

### Statistical analysis

Data are presented as the mean ± standard error of the mean. Data analysis involved one-way ANOVA followed by a least-squares difference test (with equal variances assumed) or Dunnett’s T3 test (equal variances not assumed). Pearson’s correlation was used for correlation analysis. A two-tailed *p* value of <0.05 was considered statistically significant. SPSS 18.0 was used for all statistical analyses.

## Results

After balloon injury, all rabbits showed full recovery. During the experiment, two rabbits from the high cholesterol group died of diarrhea; one at week 6 and the other at week 10. The other rabbits remained healthy throughout the study and no adverse effects were observed.

### Serum lipid assay of balloon-injured rabbits

While there were no changes in serum lipid levels of TC, LDL-C, HDL-C, and TG during weeks 4, 6, 8, 10 or 12 in control rabbits (S1 Table), rabbits from the group that received a high cholesterol diet presented with severe hypercholesterolemia and exhibited a significant increase in the levels of the four serum proteins. By week 12, the dietary cholesterol intake resulted in a marked increased in serum TC levels (3.250-fold) and LDL-C levels (4.670-fold) (both *p <* 0.05, S1 Table) compared with the serum level of TC and LDL-C at the end of week 4.

### Histopathological and IHC examination of the high cholesterol group

**Fig 1:**
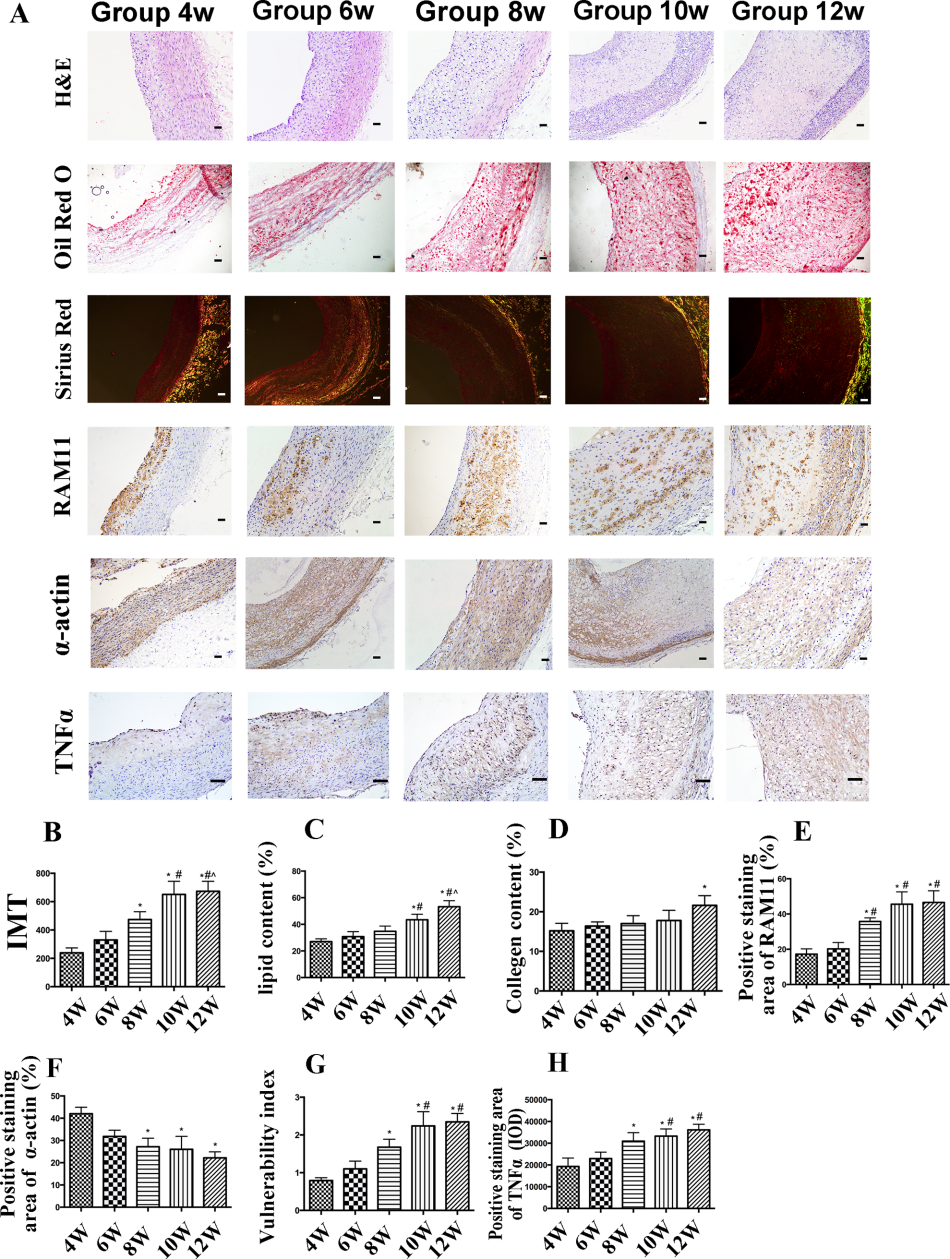
Immunohistochemical staining of plaque components in aortic plaques from high cholesterol-fed rabbits. (A) Representative cross-sectional images of the plaques in the abdominal aorta of rabbits from week 4 to 12. α-Actin staining for smooth muscle cells (SMCs) (Bar = 50 μm). RAM-11 staining for macrophages (Bar = 50 μm). Sirius red staining for collagen (Bar = 50 μm). Oil-red O staining for lipids (Bar = 50 μm). Protein expression of TNF-α (Bar = 50 μm). (B–F) Quantification of the relative expression of SMCs, macrophages, collagen, and lipid in the plaque from rabbits. (G) Vulnerability index (VI) in five groups of high cholesterol-fed rabbits from week 4 to 12. (H) Quantification of the protein expression of TNF-α. Data are presented as the mean±SEM. *p<0.05 vs. week 4; #*p*<0.05 vs. week 6; ^*p*<0.05 vs. week 8; &*p*<0.05 vs. week 10.

Few plaques were observed in control rabbits (S1 Fig). Severe hypercholesterolemia resulted in the development of atherosclerosis in the abdominal aorta in the high cholesterol group. The IMT of the abdominal aortic plaques significantly increased from week 4 to 12 in cholesterol-fed rabbits (Fig 1B).

The plaque lipid content was significantly higher at weeks 10 and 12 than at weeks 4, 6, and 8 (*p<*0.05) (Fig 1A and 1C), which did not differ among them. Positive Sirius red collagen staining was more intense in samples from week 12 than those from the other weeks (*p<*0.05), and collagen type I expression (red) significantly decreased whereas collagen III expression (yellow and green) increased from week 4 to week 12, suggesting that new collagen was synthesized whereas mature collagen was reduced with time prolonging (Fig 1A and 1D). Positive RAM-11 staining was significantly increased at weeks 8, 10, and 12 (*p<*0.05) (Fig 1A and 1E). In contrast, α-actin-positive staining in the abdominal aortic SMCs was significantly weaker at weeks 8, 10, and 12 than at weeks 4 and 6 (*p <*0.05) (Fig 1A and 1F). Therefore, compared with the VI at week 4, the VI at weeks 8, 10, and 12 gradually but significantly increased (*p*<0.05), and the VI was higher at weeks 10 and 12 than at week 6 (*p* < 0.05) (Fig 1G). To identify changes of inflammatory cytokine within the plaques, we used IHC to quantify the expression level of TNF-α. The result suggested that expression of TNF-α showed the same trend as VI (Fig 1H).

### Intraplaque neovessels are immature and leaky in the high cholesterol group

**Fig 2:**
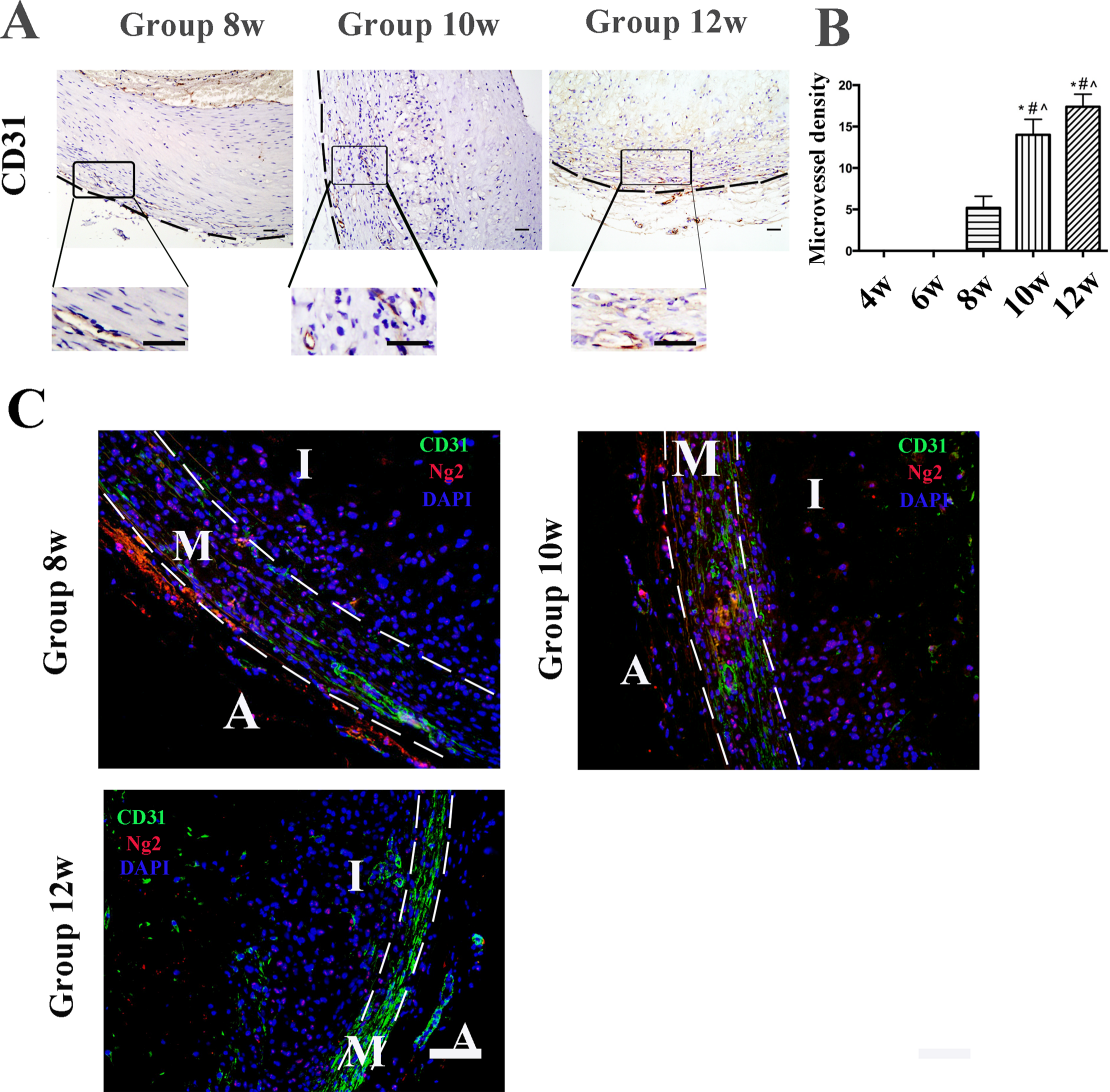
Immunohistochemical staining of CD31 and double-label immunofluorescence in aortic plaques from high cholesterol-fed rabbits. (A) Protein expression of CD31 (endothelial cell (EC) marker). (Bars = 20 μm). The lesion is outlined by the black dots. (B) Quantification of the total number of neovessels in the five groups of high cholesterol-fed rabbits from week 4 to 12. Data are presented as the mean±SEM. *p<0.05 vs. week 4; #*p*<0.05 vs. week 6; ^*p*<0.05 vs. week 8; &*p*<0.05 vs. week 10. (C) Rabbit abdominal plaque sections stained with anti-CD31 (green color, EC marker) mAb and anti-Ng2 mAb (red color, pericyte marker). Counterstained with DAPI. (Bars = 50 μm). The lesion is outlined by the white dots. *I* intima, *M* media and *A* adventitia.

Compared weeks 4 and 6, neovessels first appeared in the media of the plaque after 8 weeks of the high cholesterol diet, and as time lasted, neovessels had already extended to the inner media at week 10 and then gradually penetrated into the inner media and the pathological intima at weeks 10 and 12. Also, the number of neovessels dramatically increased from week 8 to 12 (Fig 2A and 2B, *p*<0.05). No intraplaque neovessels (as indicated by CD31) were detected in the control group (S1 Fig).

Double-label immunofluorescence (CD31 and Ng2 staining) showed that almost all observed neovessels contained only endothelial cells (ECs) (CD31) without the stabilizing layer of pericytes (Ng2), and were not dependent on the size of neovessels (Fig 2C and S2 Fig).

**Fig 3:**
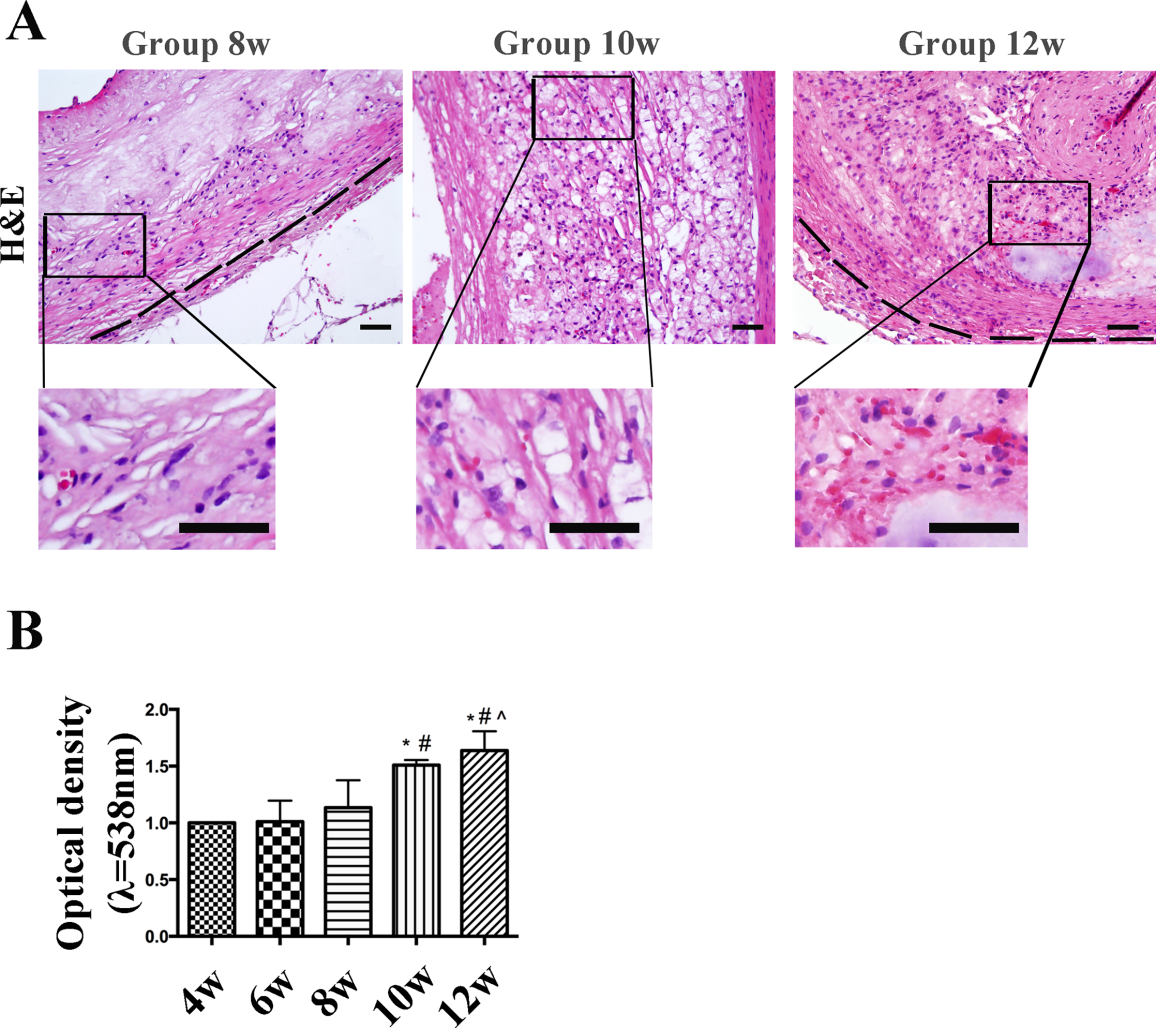
Hematoxylin & eosin (H&E) staining and FITC-dextran permeability in aortic plaques from high-cholesterol-fed rabbits. (A) H&E staining indicated the red blood cells within the plaque. (Bars = 50 μm). (B) The integrity of intraplaque neovessel endothelial barrier was evaluated by measuring FITC-dextran permeability across the endothelium. Accumulation of FITC fluorescence in the plaque was detected using a microplate reader. Data are presented as the mean±SEM from three independent experiments. *p<0.05 vs. week 4; #*p*<0.05 vs. week 6; ^*p*<0.05 vs. week 8; &*p*<0.05 vs. week 10.

Accordingly, red blood cells were in the media at week 8, and appeared in the plaque at weeks 10 and 12, and most were observed within the neovessels, as red dots (H&E staining indicated). A few red blood cells were also detected around the intraplaque neovessels (Fig 3A).

Neovessel permeability was determined by measuring FITC-dextran accumulation. There was a loss in the functional integrity of the neovessel endothelial barrier due to the lack of coverage by pericytes, as FITC-dextran permeability increased 1.673-fold in week 12 and 1.509-fold in week 10 compared with that at week 4 measured immediately after termination of irrigation (Fig 3B). Hence, the neovessels within the plaques exhibited a significant increase in the permeability rate of FITC-dextran.

### IHC examination of growth factors and their receptors in the high cholesterol group

**Fig 4:**
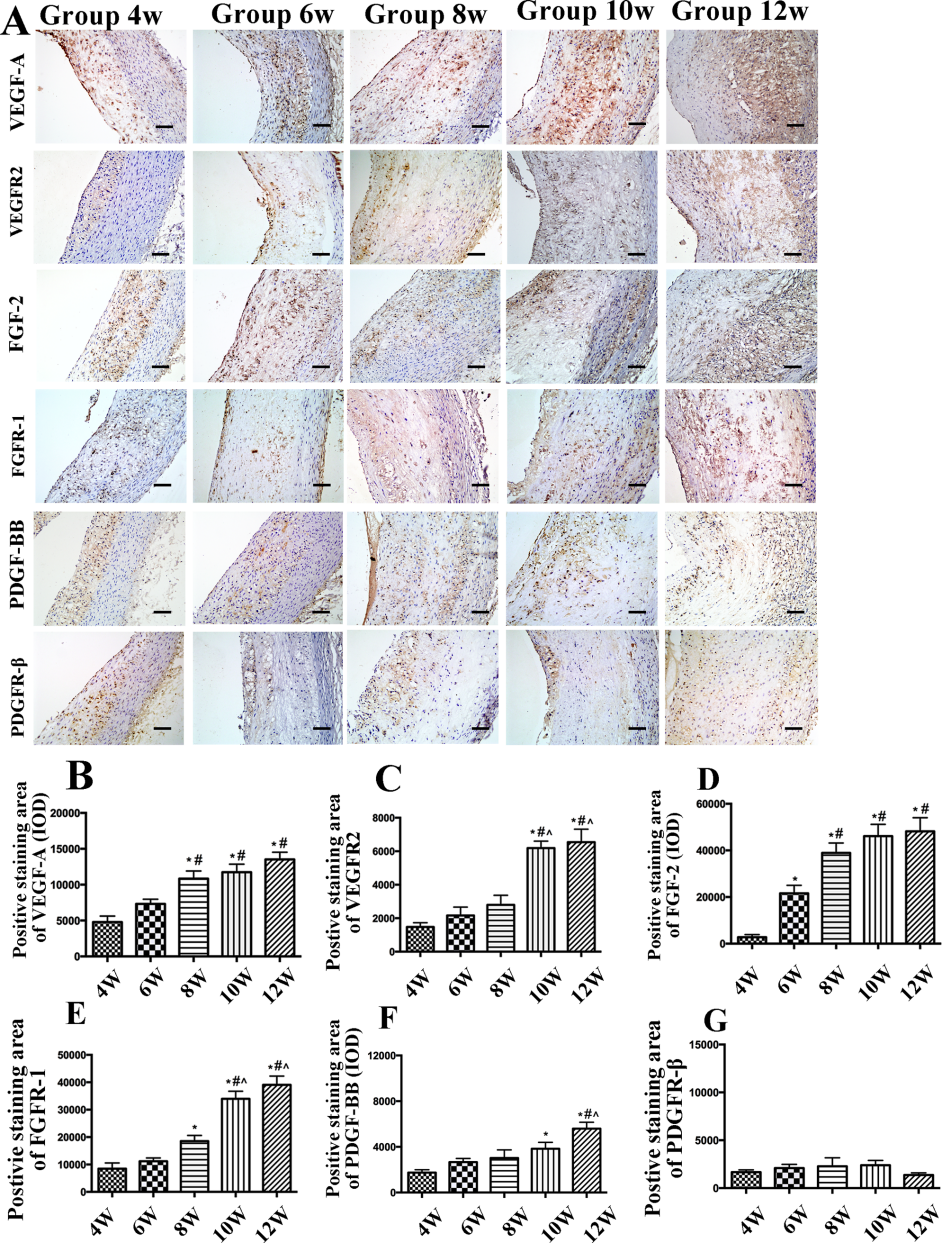
Immunohistochemical staining of growth factors and quantitative analysis in aortic plaques from high cholesterol-fed rabbits. (A) Protein expression of vascular endothelial growth factor A (VEGF-A), VEGF receptor 2 (VEGFR-2), basic fibroblast growth factor 2 (FGF-2), FGF receptor 1 (FGFR)-1, platelet-derived growth factor-BB (PDGF-BB) and PDGF receptor β (PDGFR-β). (Bars = 50 μm). (B–F) Quantification of VEGF-A, VEGFR-2, FGF-2, FGFR-1, PDGF-BB, and PDGFR-β. Data are presented as the mean±SEM. *p<0.05 vs. week 4; #*p*<0.05 vs. week 6; ^*p*<0.05 vs. week 8; &*p*<0.05 vs. week 10.

Staining of TNF-α, growth factors and their receptors was rarely observed in ECs, macrophages, or SMCs from the control group (S1 and S3 Figs). In contrast, in the high cholesterol group, the relative expression of TNF-α, VEGF-A, VEGFR-2, FGF-2, FGFR-1, and PDGF-BB in plaques exhibited the same increased trend as VI (Fig 1H and Fig 4A–4F).

Moreover, while expression of these factors significantly increased from week 4 to 12, the extent of PDGFR-β-positive staining did not differ from week 4 to 12 (Fig 4G). Compared with those in the control group, the relative expression levels of VEGF-A and FGF-2 at week 4 were 11.644- and 4.607- fold, respectively. However, at week 6, the relative expression of FGF-2 increased more quickly than that of VEGF-A, and the relative expression levels of VEGF-A and FGF-2 in the high cholesterol group at week 12 were respectively 30.814- and 67.638- folds higher than those in the control group (S2 Table).

**Fig 5:**
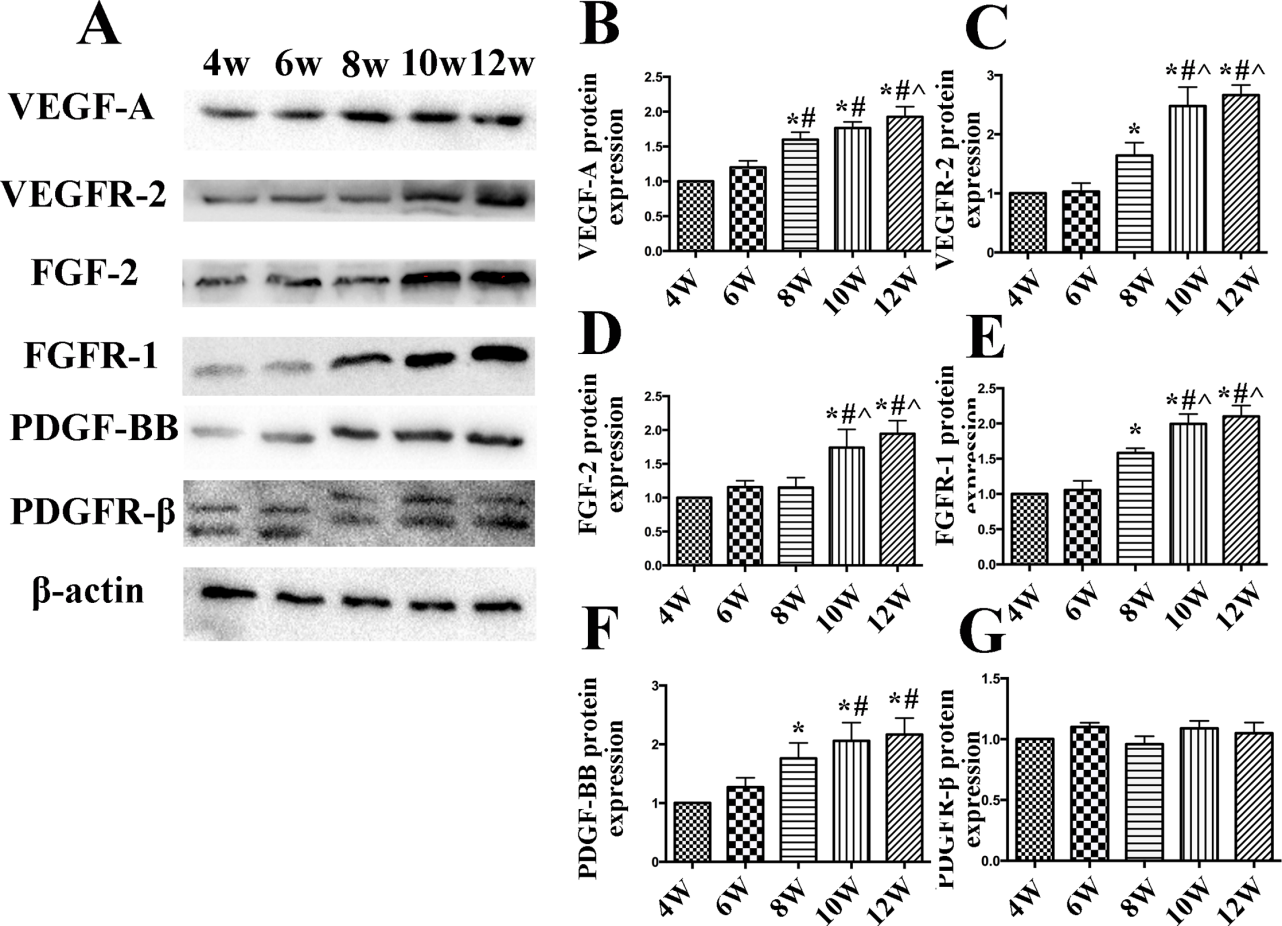
Western blot analysis and quantification of growth factors in aortic plaques from high cholesterol-fed rabbits. (A) Protein expression of vascular endothelial growth factor A (VEGF-A), VEGF receptor 2 (VEGFR-2), basic fibroblast growth factor 2 (FGF-2), FGF receptor 1 (FGFR)-1, platelet-derived growth factor-BB (PDGF-BB) and PDGF receptor β (PDGFR-β). (B-G) Quantification of VEGF-A, VEGFR-2, FGF-2, FGFR-1, PDGF-BB, and PDGFR-β. Each experiment was repeated three times. Data are presented as the mean±SEM. *p<0.05 vs. week 4; #*p*<0.05 vs. week 6; ^*p*<0.05 vs. week 8; &*p*<0.05 vs. week 10.

**Fig 6:**
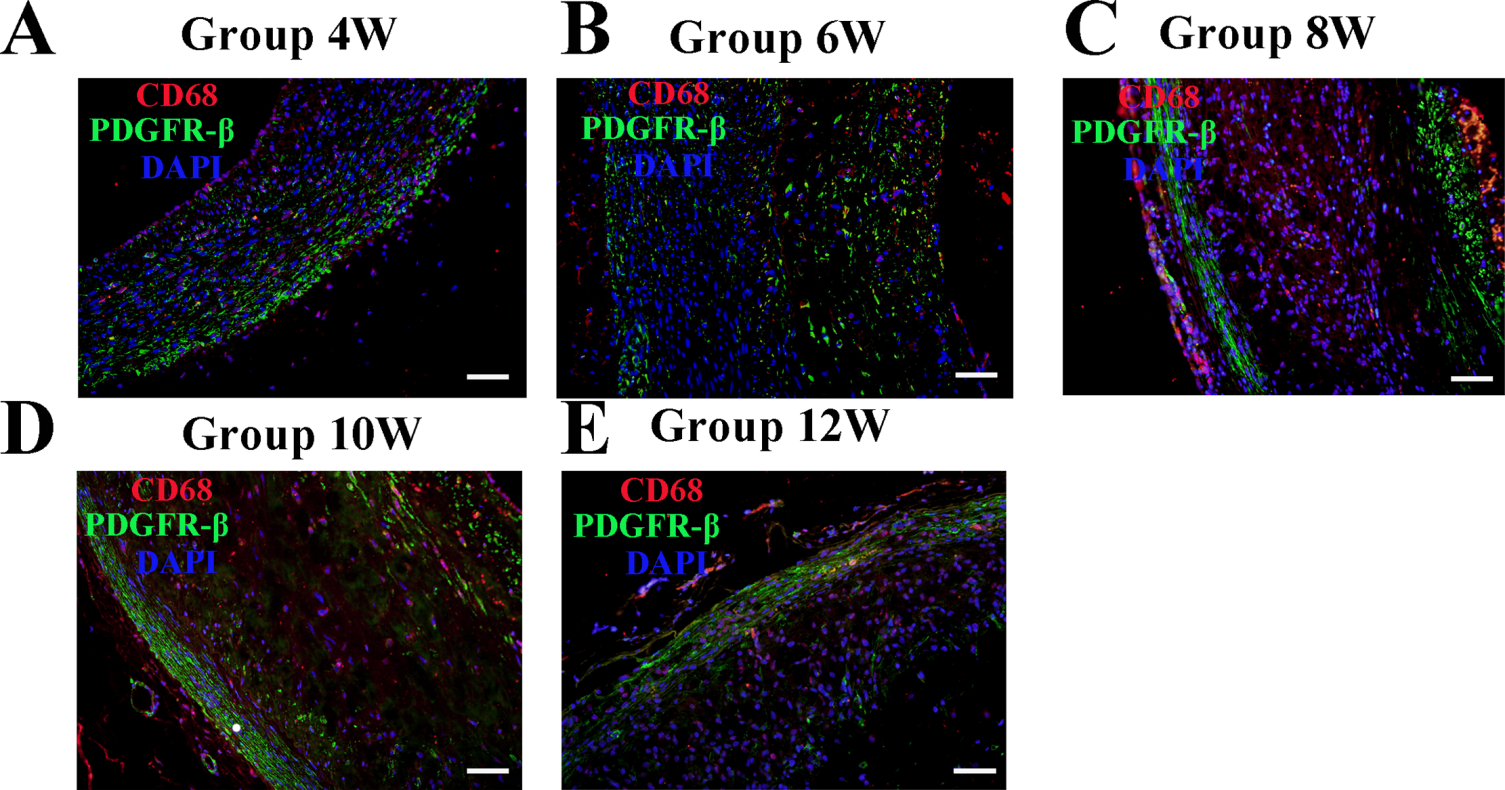
Co-localization of PDGF receptorβ (PDGFR-β) and of CD68-expressing cells in aortic plaques from high-cholesterol-fed rabbits. (A-E) Rabbit abdominal plaque sections stained with anti-PDGFR-β mAb (green color) and anti-CD68 (red color) mAb (Bars = 50 μm). Counterstained with DAPI.

Western blot analysis in the high cholesterol group indicated that the expression of VEGF-A, VEGFR-2, FGF-2, FGFR-1, PDGF-BB, and PDGFR-β exhibited the same trends as those shown in the IHC results (Fig 5A–5G). Double-label immunofluorescence indicated that PDGFR-β expression did not co-localize with foam cells (as indicated by CD68) (Fig 6A–6E).

### Correlation analysis in the high cholesterol group

Correlation analysis results in the high-cholesterol-fed animals are shown in Table 1. All correlations between the VI and the levels of LDL-C, TNF-α, VEGF-A, VEGFR-2, FGF-2, FGFR-1, and PDGF-BB were positive (r = 0.613, 0.843, 0.498, 0.674, 0.644, 0.737, 0.640, and 0.691 respectively; all *p*<0.05). The correlation between TNF-α and VEGF-A, VEGFR-2, FGF-2, FGFR-1, and PDGF-BB were all positive (r = 0.447, 0.509, 0.746, 0.504, and 0.465; all *p<*0.05, respectively). The expression of VEGF-A, VEGFR-2, FGF-2, FGFR-1, and PDGF-BB was positively correlated with the MVD in plaques (r = 0.692, 0.787, 0.720, 0.851, and 0.623, respectively; all *p*<0.05). However, there was no correlation between PDGFR-β expression and the MVD in plaques (r = -0.030, *p*>0.05).

**Table 1:** Pearson’s correlations between angiogenic growth factors levels, the vulnerability index (VI), microvascular density (MVD), total cholesterol (TC), low-density lipoprotein cholesterol (LDL-C), RAM11, and TNFα in balloon-injured rabbits.

	VEGF-A	FGF-2	PDGF-BB	VEGFR-2	FGFGR-1	PDGFR-β	RAM11	TNFα	LDL-C
**VI**	0.674^a^	0.737^a^	0.691^a^	0.644^a^	0.640^a^	0.061	0.704^a^	0.498^b^	0.613^a^
**MVD**	0.692^a^	0.720^a^	0.623^a^	0.787^a^	0.851^a^	-.030	0.753^a^	0.700^a^	0.768^a^
**VEGF-A**	1.000	0.700^a^	0.641^a^	0.682^a^	0.762^a^	0.116	0.638^a^	0.674^a^	0.776^a^
**LDL-C**	0.776^a^	0.641^a^	0.577^a^	0.823^a^	0.838^a^	0.030	0.648^a^	0.529^a^	1.000
**RAM11**	0.638^a^	0.691^a^	0.478^b^	0.547^a^	0.729^a^	-.024	1.000	0.636^a^	0.648^a^
**TNFα**	0.447^b^	0.746^a^	0.465^b^	0.509^a^	0.504^b^	-.268	0.636^a^	1.000	0.529^a^

^a^Statistically significant, *p* < 0.01

^b^Statistically significant, *p* < 0.05.

VEGF-A, vascular endothelial growth factor-A; VEGFR-2, vascular endothelial growth factor receptor 2; FGF-2, fibroblast growth factor-2; FGFR-1, fibroblast growth factor receptor 1; PDGF-BB, platelet-derived growth factor-BB; PDGFR-β, platelet-derived growth factor receptor β, TNFα, tumor necrosis factor

## Discussion

The present study demonstrated that hypercholesterolemia increased the release of inflammatory cytokine TNF-α and then promoted intraplaque angiogenesis by enhancing VEGF-A/VEGFR-2 and FGF-2/FGFR-1 expression. However, PDGF-BB/PDGFR-β may not play a role in this process. Therefore, the immature and inflammatory neovessels within the plaque accelerated the progression and instability of AS lesions in rabbits. For all we know, this is the first longitudinal study on the interaction among neovessels, inflammation and plaque vulnerability, which demonstrate the process of vasa vasorum extension and penetration, verify the increased permeability of neovessel, and elucidate that the different growth factors might play different roles in the different stages of atherosclerosis and plaque vulnerability.

Atherosclerosis is a multifocal alteration of the vascular wall of medium and large arteries that is characterized by local accumulation of cholesterol and non-resolving inflammation. A critical step in atherogenesis is the early recruitment of inflammatory monocytes and their differentiation into foam macrophages in the nascent plaque; the mechanism of lipid uptake initiating monocyte activation has previously been clearly defined [10, 11]. As demonstrated in our study, Pearson’s correlation indicated that dramatically elevated levels of LDL-C were associated with the expression of the inflammatory factor TNF-α (r = 0.648, *p* < 0.05) and the accumulation of inflammatory cell macrophages (r = 0.529, *p* < 0.05) within the plaque. Hyperlipidemia leads to the increased release of TNF-α and macrophages accumulation in plaques. By acting synergistically, inflammation might in turn stimulate further lipid retention [12]. An increasing focus on TNF-α-induced angiogenesis has been implicated not only in atherosclerotic plaque but also in the progression of cancer, wet age-related macular degeneration and rheumatoid arthritis [13, 14]. In the present study, we found positive correlations between TNF-α and MVD, and thus, TNF-α might also be involved in the process of intraplaque angiogenesis, which promotes plaque development and instability.

Intraplaque neovascularization sprouting from the adventitial vasa vasorum has been identified as an independent predictor of intraplaque hemorrhaging and plaque rupture [15]. Based on the studies on carotid endarterectomy, intraplaque angiogenesis has been recognized as not only a prominent feature of advanced AS lesions but also a driving force of plaque rupture and subsequent thrombotic events. The present study showed the process of vasa vasorum extension and penetration that neovessels first appear in the media at week 8, and as time lasted, neovessels gradually penetrated into the plaque at weeks 10 and 12.

Chondroitin Sulfate Proteoglycan Ng2 is a well-established pericyte marker. In this study, the negative staining of Ng2 demonstrated that those neovessels (positive CD31 staining, green) were immature because of lacking the circle of pericytes and vascular smooth muscle cells (negative Ng2 staining, red). These microvessels were found to be susceptible to leakage, which allowed inflammatory cells and erythrocytes to easily infiltrate the neovessels and accumulate deep within the plaque [16]. Our findings demonstrated that the permeability rate of FITC-dextran significantly increased from week 4 to 12, and H&E staining indicated that there were red blood cells permeated from the leaky neovessels. Red blood cell membrane contributes to cholesterol accumulation within the plaques by increasing free cholesterol in the plaque. The present study showed that there were high correlations between the MVD and the IMT and VI of plaque. Thus, inflammatory and immature neovascularization might also be lead to the progression and destabilization of AS plaques.

An important finding in this study was that multiple angiogenic factors might be involved in the process of immature and inflammatory intraplaque neovessels. The angiogenic process is mediated by classical angiogenic factors and by additional AS angiogenesis-specific factors [17].

The vascular endothelial growth factors (VEGFs) and their tyrosine kinase receptors are the main pathway involved in angiogenesis and stimulate angiogenesis by increasing the proliferation, migration, and permeation of ECs, and by enhancing vascular permeability [18]. A previous study revealed that VEGF-A expression alone initially leads to vessel enlargement followed by a loss of pericytes [19]. A high cholesterol diet might increase the expression of serum VEGF-A in nephropathia epidemica [20]. Our study indicated that VEGF-A and VEGFR-2 expression levels dramatically increased from week 4 to 12, and the correlation between LDL-C and VEGF-A/VEGFR-2 was positive. Studies showed that TNF-α was previously shown to induce VEGF and FGF expression in different vascular cell types and to require VEGF and FGF for optimal stimulation of angiogenesis [21, 22]. Therefore, in the present study, inflammation might be another cause of the release of VEGF-A and for the positive relationship between TNF-α and VEGF-A/VEGFR-2, FGF-2/FGFR-1.

Although FGF-2 and PDGF-BB are thought to be important regulators of plaque angiogenesis, their precise roles in regulating and coordinating this complex process in intraplaque angiogenesis remain unknown. Both FGF-2 and PDGF-BB have been called “indirect” angiogenic growth factors because they stimulate angiogenesis in vivo but fail to demonstrate mitogenic activity for ECs in vitro. Previous studies showed that FGF-2/FGFR-1 is critical for forming luteal endothelial networks and provides a pattern for the vasa vasorum to form a plexus-like network [23, 24, 25]. There is increasing evidence that the FGF system is positioned upstream of more specialized growth factor systems for ECs [26], because addition of exogenous FGF-2 or up-regulation of endogenous FGF-2 expression increases VEGF synthesis; however, quiescent ECs do not express VEGF-A in vitro [27]. In our study, there were positive correlations between FGF-2/FGFR-1 and VEGF-A/VEGFR-2. Thus, FGF-2/FGFR-1 might induce the expression of VEGF-A/VEGFR-2 in the plaque and then generate a platform for intraplaque angiogenesis.

Another important finding in this study was that different from VEGF-A/VEGFR-2 and FGF-2/FGFR-1, the PDGF-BB/PDGFR-β signaling pathway was not involved in angiogenesis in the advanced plaque stage. The interaction between PDGF-BB and PDGFR-β is mediated by the interaction between ECs and pericytes, which induces vascular SMC (VSMCs) and pericyte proliferation, and enhances migration in vitro, thereby maintaining homoeostasis and promoting blood vessel maturation [28,29,30]. These studies demonstrate the importance of the interaction between PDGF-BB and PDGFR-β in mediating pericyte coverage. In this study, although the expression levels of PDGF-BB in plaques was relatively high, the proportion of the PDGFR-β-positive area did not differ from week 4 to 12, which indicated that PDGF-BB/PDGFR-β may not play an active role in intraplaque angiogenesis at the advanced plaque stage.

Moreover, VEGF-A has been shown to negatively regulate pericyte function by inhibiting PDGFR-β phosphorylation through the formation of a nonfunctional VEGFR-2/PDGFR-β complex, which results in a reduction in vascular pericyte coverage [31]. VEGF-A plays a major role in the earlier stage of intraplaque angiogenesis, during which PDGFR-β expression was diminished, leading to an almost complete loss of pericytes. In this study, the high expression of VEGF-A/VEGFR-2 and FGF-2/FGFR-1 and maintained low expression of PDGFR-β from week 4 to 12 were regarded as the leading cause of immature and inflammatory intraplaque neovessels and plaque instability.

Previous study indicated that oxidized low-density lipoproteins (oxLDLs) induce a dual effect on PDGFR-β signaling. Short-term incubation of SMCs with oxLDLs induced PDGFR-β activation, while long-term incubation inhibited its phosphorylation and increased the formation of 4-hydroxynonenal (4-HNE)-PDGFR adducts in SMCs [32]. This effect may be related to the fact that PDGFR-β did not co-localize with foam cells (CD68). Thus, hypercholesterol may have a direct role in the consistent expression of PDGFR-β in the abdominal aortic plaque.

Some limitations need to be acknowledged; first, the sample size was relatively small. In the future, further studies with larger sample sizes are warranted. Second, extensive studies should be undertaken to fully elucidate the biochemical and cellular mechanisms.

Taken together, the results indicated that hypercholesterolemia-increased the accumulation of foam cells and the release of TNF-α and VEGF-A. Hypercholesterolemia, inflammation, and multiple growth factors, including VEGF-A/VEGFR-2 and FGF-2/FGFR-1 but not PDGF-BB/PDGFR-β might act synergistically to promote the development of immature and inflammatory intraplaque neovessels, and to accelerate the progression and instability of AS plaques in rabbits. This study may provide useful information for choosing time of intervention before targeted drug or gene therapy on intraplaque neovessels as well.

## Supporting information

S1 Fig Hematoxylin & eosin (H&E), α-actin, RAM-11, TNF-α, and CD31 staining in the abdominal aorta of rabbits in the control group. (A-C) Only fatty streaks with lipid infiltration were present in the abdominal aorta of rabbits in the control group. Positive staining was rare TNF-α and absent for neovessels (CD31 indicated) in the plaque of the control group. (Bars = 50 μm).
(TIF)

S2 Fig Double-label immunofluorescence of CD31 and Ng2 in aortic plaques from high cholesterol-fed rabbits from week 4 and week 6. (A-B) Rabbit abdominal plaque sections stained with anti-CD31 (green color, EC marker) mAb and anti-Ng2 mAb (red color, pericyte marker). Counterstained with DAPI. (Bars = 50 μm). The lesion is outlined by the white dots. *I* intima, *M* media and *A* adventitia.
(TIF)

S3 Fig Immunohistochemical (IHC) staining of growth factors in the abdominal aorta in the control group. (A-G) Positive staining for vascular endothelial growth factor A (VEGF-A), VEGF receptor 2 (VEGFR-2), basic fibroblast growth factor 2 (FGF-2), FGF receptor 1 (FGFR)-1, platelet-derived growth factor-BB (PDGF-BB) and PDGF receptor β (PDGFR-β) were rarely observed in the control group. (Bars = 50 μm).
(TIF)

S1 Table Serum lipid profiles (mmol/L). (DOCX)

S2 Table The comparison of relative expression of VEGF-A, VEGFR-2, FGF-2, and FGFR-1 between hypercholesterol groups and the control group (folds). (DOCX)
