# Supplementary figures and images for: VEGF-A/VEGFR-2 and FGF-2/FGFR-1 but not PDGF-BB/PDGFR-β play important roles in promoting immature and inflammatory intraplaque angiogenesis
Source: PLoS One. 2018 Aug 20;13(8):e0201395. doi: 10.1371/journal.pone.0201395 (PMC6101364; doi:10.1371/journal.pone.0201395)

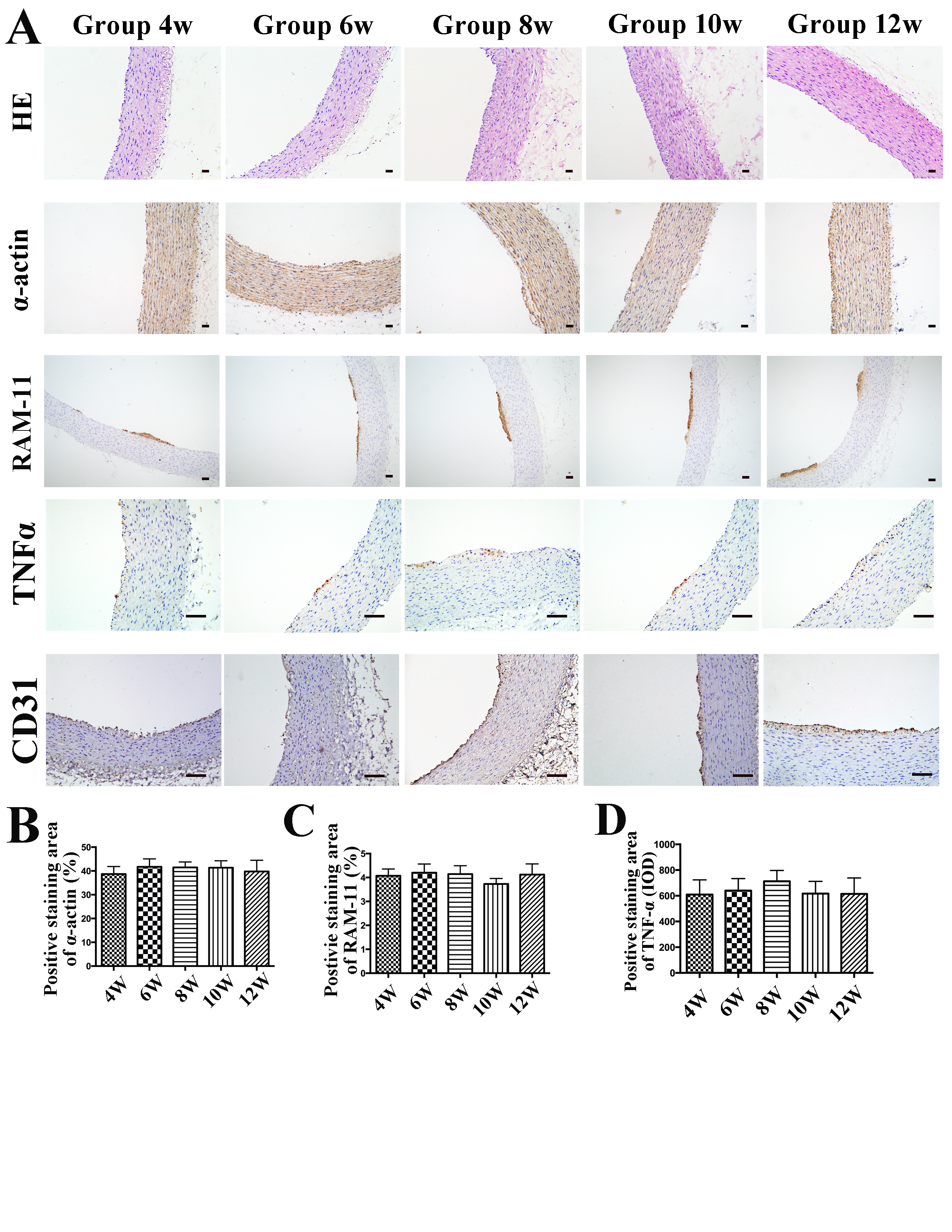

Supplement: S1 Fig — (A-C) Only fatty streaks with lipid infiltration were present in the abdominal aorta of rabbits in the control group. Positive staining was rare TNF-α and absent for neovessels (CD31 indicated) in the plaque of the control group. (Bars = 50 μm). (TIF) [file pone.0201395.s001.tif]

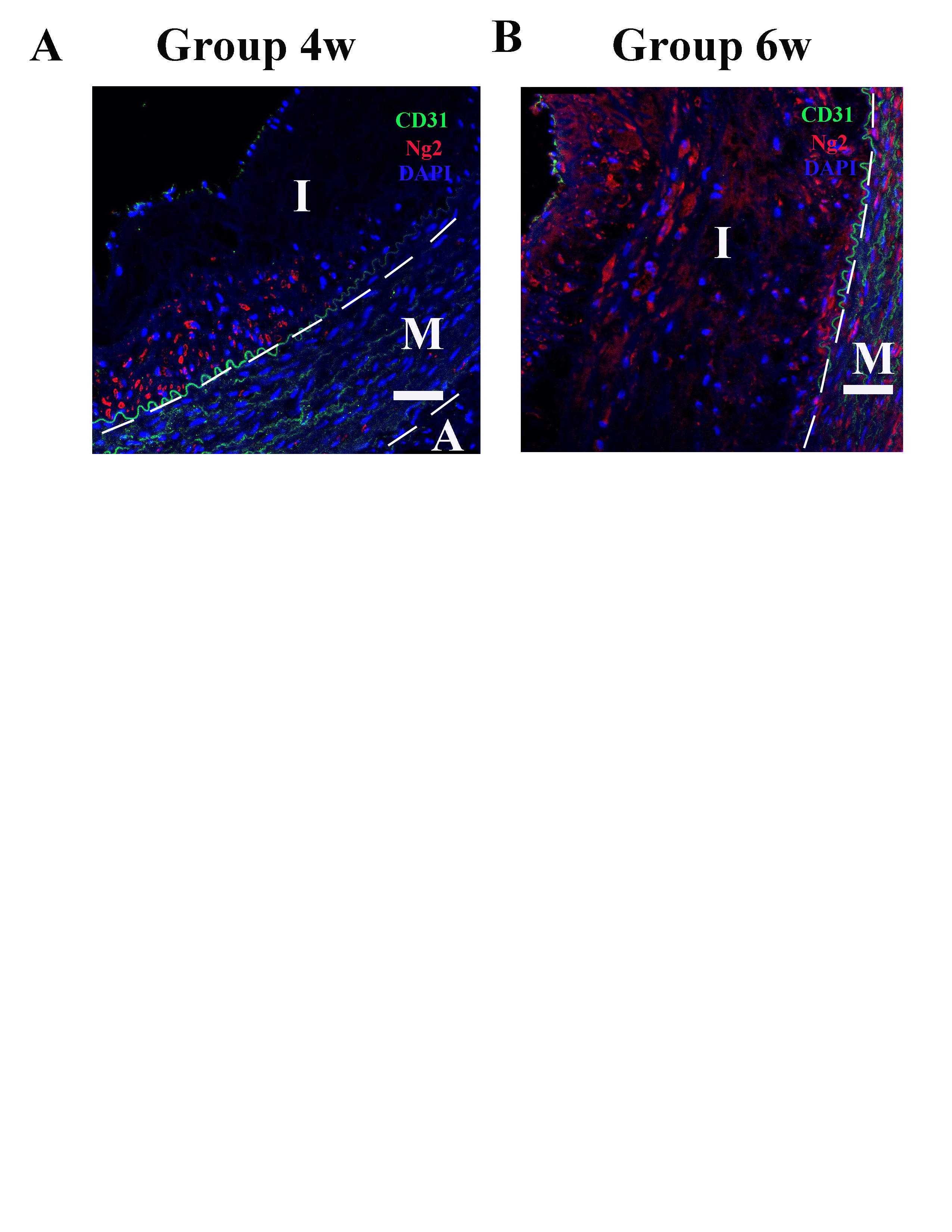

Supplement: S2 Fig — (A-B) Rabbit abdominal plaque sections stained with anti-CD31 (green color, EC marker) mAb and anti-Ng2 mAb (red color, pericyte marker). Counterstained with DAPI. (Bars = 50 μm). The lesion is outlined by the white dots. I intima, M media and A adventitia. (TIF) [file pone.0201395.s002.tif]

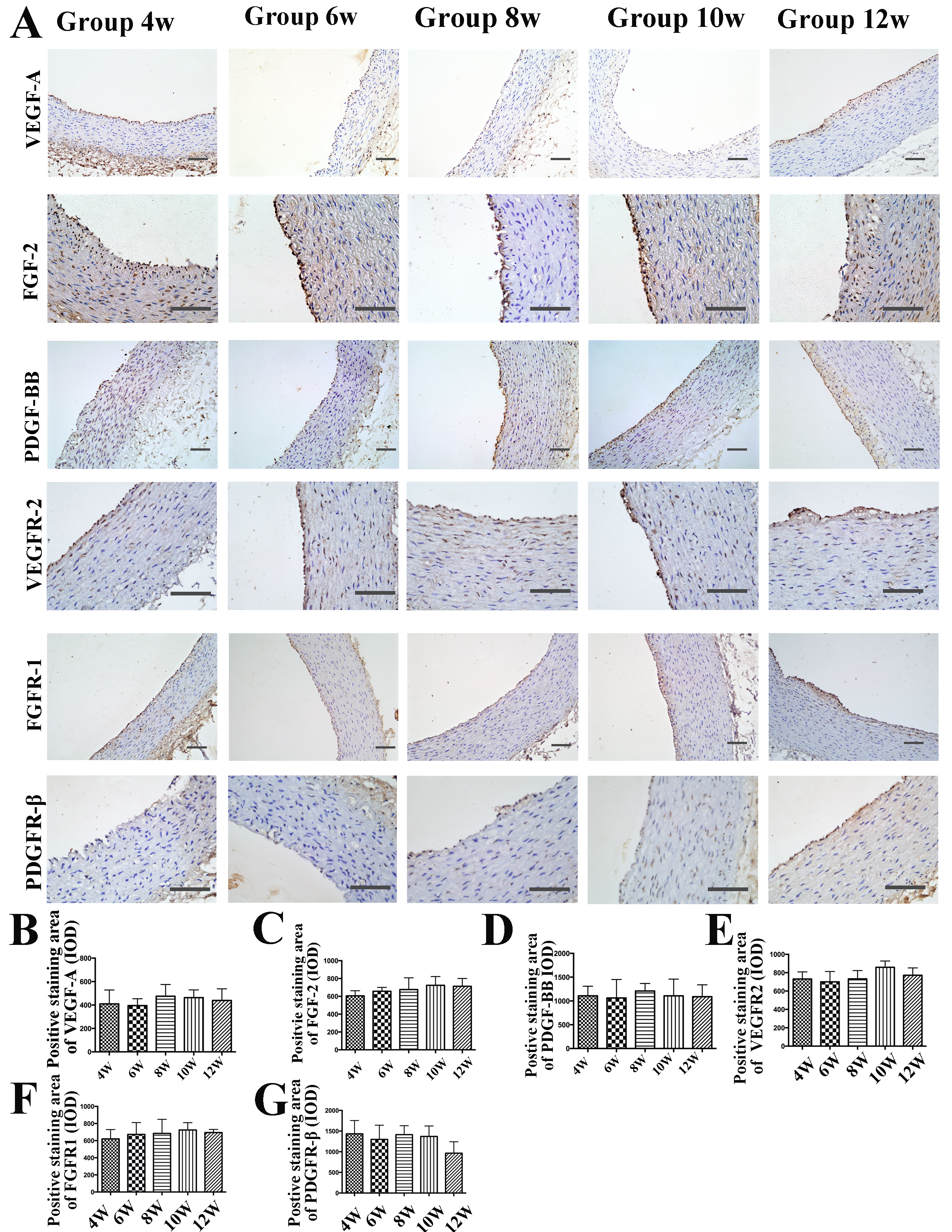

Supplement: S3 Fig — (A-G) Positive staining for vascular endothelial growth factor A (VEGF-A), VEGF receptor 2 (VEGFR-2), basic fibroblast growth factor 2 (FGF-2), FGF receptor 1 (FGFR)-1, platelet-derived growth factor-BB (PDGF-BB) and PDGF receptor β (PDGFR-β) were rarely observed in the control group. (Bars = 50 μm). (TIF) [file pone.0201395.s003.tif]
