# Supplementary material for: VEGF-A/VEGFR-2 and FGF-2/FGFR-1 but not PDGF-BB/PDGFR-β play important roles in promoting immature and inflammatory intraplaque angiogenesis
Source: PLoS One. 2018 Aug 20;13(8):e0201395. doi: 10.1371/journal.pone.0201395 (PMC6101364; doi:10.1371/journal.pone.0201395)
Supplement: S1 Table — (DOCX) [file pone.0201395.s004.docx]

**Supplement Table 1.** Serum lipid profiles (mmol/L)

|  | 4W | 6W | 8W | 10W | 12W |
| --- | --- | --- | --- | --- | --- |
| HDL-C_control_ | 1.395±0.326 | 1.317±0.184 | 1.344±0.291 | 1.268±0.233 | 1.289±0.253 |
| LDL-C_control_ | 2.147±0.411 | 2.174±0.332 | 2.137±0.336 | 2.223±0.472 | 2.151±0.428 |
| TC_control_ | 8.356±1.439 | 9.608±2.524 | 10.512±1.439 | 9.798±3.379 | 9.374±1.843 |
| TG_control_ | 0.748±0.093 | 0.692±0.121 | 0.686±0.118 | 0.730±0.082 | 0.751±0.073 |
| HDL-C | 1.236±0.181 | 2.516±0.719 | 2.936±0.798 | 3.368±0.689 | 3.652±0.677 |
| LDL-C | 2.172±1.171 | 4.496±1.594 | 6.344±1.969 | 9.520±2.273 | 10.144±2.947 |
| TC | 12.156±4.139 | 22.676±7.019 | 26.444±6.604 | 35.998±6.799 | 39.514±8.223 |
| TG | 0.660±0.176 | 0.954±0.211 | 1.186±0.238 | 1.410±0.262 | 1.574±0.398 |

Data are expressed as mean±SEM; HDL-C: high-density lipoprotein cholesterol; LDL-C: low-density lipoprotein cholesterol; TC: total cholesterol; TG: triglyceride.
