# Supplementary material for: VEGF-A/VEGFR-2 and FGF-2/FGFR-1 but not PDGF-BB/PDGFR-β play important roles in promoting immature and inflammatory intraplaque angiogenesis
Source: PLoS One. 2018 Aug 20;13(8):e0201395. doi: 10.1371/journal.pone.0201395 (PMC6101364; doi:10.1371/journal.pone.0201395)
Supplement: S2 Table — (DOCX) [file pone.0201395.s005.docx]

**Supplement Table 2.** The comparison of relative expression of VEGF-A, VEGFR-2, FGF-2, and FGFR-1 between hypercholesterol groups and the control group (folds).

|  | 4W | 6W | 8W | 10W | 12W |
| --- | --- | --- | --- | --- | --- |
| VEGF-A | 11.644 | 18.555 | 22.831 | 25.368 | 30.814 |
| VEGFR2 | 2.018 | 3.091 | 3.819 | 7.204 | 8.468 |
| FGF-2 | 4.607 | 32.846 | 57.549 | 63.729 | 67.638 |
| FGFR1 | 13.655 | 16.589 | 27.092 | 48.848 | 56.228 |

VEGF-A, vascular endothelial growth factor-A; VEGFR-2, vascular endothelial growth factor receptor 2; FGF-2, fibroblast growth factor-2; FGFR-1, fibroblast growth factor receptor 1.
